# A Study on the Photoisomerization of (*E*)-Dehydrozingerone, Its (*E*)-(*E*)-C₂ Symmetric Dimer, and Their *O*-Methylated Derivatives

**DOI:** 10.3390/molecules29245901

**Published:** 2024-12-13

**Authors:** Maria Antonietta Dettori, Valeria Ugone, Davide Fabbri, Paola Carta

**Affiliations:** Istituto di Chimica Biomolecolare, Consiglio Nazionale delle Ricerche, Traversa La Crucca 3, I–07100 Sassari, Italy; mariaantonietta.dettori@cnr.it (M.A.D.); valeria.ugone@cnr.it (V.U.); paola.carta@cnr.it (P.C.)

**Keywords:** curcumin analogs, photoisomerization, NMR spectroscopy, UV light exposure, hydroxylated biphenyls, UV-Vis spectroscopy, in silico studies

## Abstract

In this study, UV-induced (*E*)-to-(*Z*) geometrical isomerizations of the curcumin degradation product (*E*)-dehydrozingerone, along with curcumin-inspired (*E*)-*O*-methylated dehydrozingerone and their corresponding C_2_-symmetric dimers, were investigated. All compounds produced corresponding (*Z*) isomers in varying yields upon UV irradiation in deuterated solvents. The efficiency of these photoisomerizations depended on the solvent and wavelength used. While (*Z*) dehydrozingerone and its corresponding (*Z*)-(*Z*) dimer proved to be highly unstable during purification, the *O*-methylated derivatives were successfully isolated, fully characterized by NMR spectroscopy, and further analyzed by UV-Vis spectroscopy and computational methods.

## 1. Introduction

**Figure 1:**
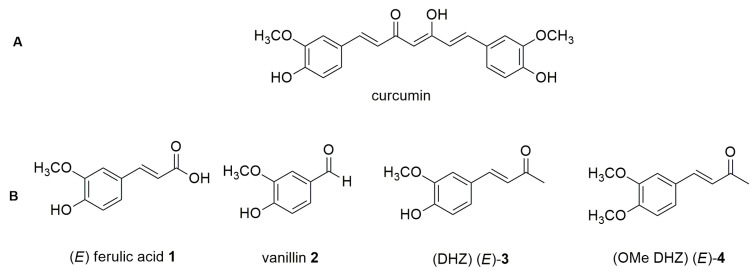
Chemical structures of curcumin (**A**); (*E*)-ferulic acid **1**, vanillin **2**, DHZ (*E*)-**3,** and OMe DHZ (*E*)-**4** (**B**).

Curcumin, or diferuloylmethane (Figure 1A), a polyphenol extracted from the rhizome of Curcuma Longa, has a rich history of use in traditional Chinese medicine [1]. Its versatility lies in its ability to influence various biological targets within human physiology, endowing it with significant anti-cancer [2], anti-inflammatory [3], and anti-oxidant [4] properties. As a member of the natural polyphenol class, curcumin is frequently employed to stave off cardiovascular and neurodegenerative diseases and to combat bacterial and viral infections [5]. However, despite its myriad benefits, curcumin is plagued by significant limitations for use in medicine due to its rapid autoxidative degradation at physiological pH. This process leads to the formation of several byproducts, including (*E*) ferulic acid **1**, vanillin **2**, and dehydrozingerone (DHZ) (*E*)-**3** [6] (Figure 1B). In our previous studies, we synthesized compound (*E*)-**3** following the classic Claisen–Schmidt condensation reaction and its derivative *O*-methyl dehydrozingerone (OMe DHZ) (*E*)-**4** through an innovative one-pot, two-step sequential methodology assisted by microwave irradiation [7].

In the Claisen–Schmidt condensation reaction, the formation of a double bond leads to the possibility of two isomers: (*E*) and (*Z*). The (*E*)-isomer, where the bulky substituents are positioned on opposite sides of the double bond, is thermodynamically more stable than the (*Z*)-isomer, where these substituents are positioned on the same side. This is because in the (*E*) configuration, the steric hindrance between the large groups is minimized, thereby reducing repulsive interactions. The resulting geometric features likely play a key role in the compound’s biological activities. Both compounds (*E*)-**3** and (*E*)-**4**, containing an α-β-unsaturated ketone chain and a guaiacyl moiety, share structural similarities with curcumin but often exhibit improved bioavailability and stability. In our previous studies, we demonstrated that compounds (*E*)-**3** and (*E*)-**4** displayed notable activity as inhibitors of α-synuclein aggregation [8], as antioxidants [9], and as antitumoral agents [10]. These findings underscore the potential of these compounds as promising candidates for therapeutic interventions, offering enhanced efficacy, stability, and cellular uptake compared to curcumin.

**Figure 2:**
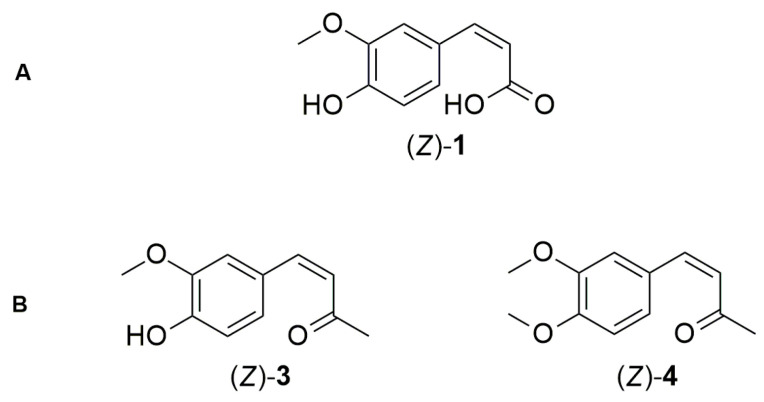
Chemical structures of ferulic acid (*Z*)-**1** (**A**); DHZ (*Z*)-**3**, and OMe DHZ (*Z*)-**4** (**B**).

The UV-induced (*E*)-to-(*Z*) geometrical isomerization of olefins is among the most thoroughly researched photochemical reactions [11]. Photoisomerization via intramolecular triplet sensitization is a well-established process [12]. (*E*) ferulic acid **1**, a secondary metabolite belonging to the hydroxyl-cinnamic acid class, is found in numerous plants and is known for its anti-inflammatory, cardioprotective, neuroprotective, and anti-carcinogenic properties [13]. Numerous studies have examined the photocatalyzed isomerization of (*E*) ferulic acid **1** to its (*Z*)-**1** isomer (Figure 2A) [12]. Cinnamic acids are usually bio-synthesized in (*E*) configuration, but UV light exposure triggers geometric isomerization, forming unnatural (*Z*) isomers [14,15]. In some cases, the (*Z*) form of cinnamic acids exhibits higher bioactivity than their corresponding (*E*) isomers [16].

Ferulic acid (*E*)-**1** and dehydrozingerone (*E*)-**3** share structural similarities, including an unsaturated chain at the para position relative to the phenolic hydroxyl, conjugated with the aromatic guaiacyl system. However, dehydrozingerone features a ketone at the terminal end of this chain, while ferulic acid has a carboxyl group in the same position. These differences may significantly influence the reactivity of the two molecules during (*E*)-to-(*Z*) photoisomerization. To the best of our knowledge, no synthetic method has yet been reported for obtaining α,β-unsaturated carbonyl compounds (*Z*)-**3** and (*Z*)-**4** (Figure 2B), neither from their isomers (*E*)-**3** and (*E*)-**4** nor via alternative synthetic routes. The synthesis of these molecules with (*Z*) geometry could result in structures with higher bioactivity than their (*E*) isomer counterparts, as suggested by studies reporting the greater bioactivity of *Z*-isomers in cinnamic acids [17]. During studies on the photochemical behavior of α,β-unsaturated ketones, Moni et al. [18] detected no isomerization of compound (*E*)-**3** at all, neither at 300 nm nor 350 nm in deuterated methanol as the solvent.

**Figure 3:**
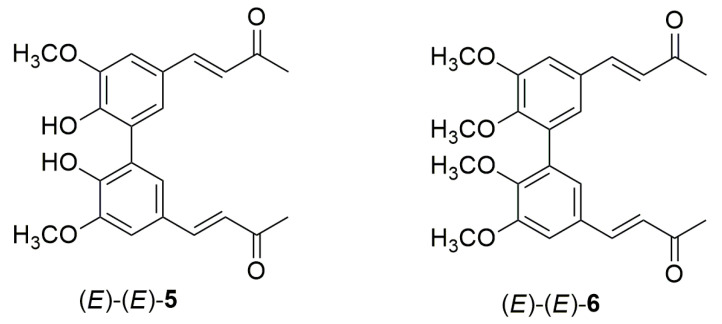
Chemical structures of compounds (*E*)-(*E*)-**5** and (*E*)-(*E*)-**6**.

C_2_-symmetric hydroxylated biphenyls are found in several natural molecules and often play a significant role in various biological structures. These compounds represent the main components of biologically relevant natural substances such as ellagitannins and lignins [19]. The chemical structure of hydroxylated biphenyls, characterized by two phenyl rings joined by a single carbon–carbon bond, induces particular effectiveness in specific ligands for receptor molecules and could be used to design new drugs for innovative therapies [19]. Generally, hydroxylated biphenyls show superior biological activities compared to their corresponding monomers [20]. For many years, our group has been interested in synthesizing variously substituted, natural-like hydroxylated biphenyls and assessing their biological activities. Notably, our previous studies have highlighted that compound (*E*)-(*E*)-**6**, the C_2_ dimer of (*E*)-**4**, is more effective in inhibiting cell proliferation and inducing apoptosis in various melanoma cell lines than its monomer and curcumin itself [21]. Additionally, compound (*E*)-(*E*)-**5**, the C_2_ dimer of (*E*)-**3**, proved to act as an effective chain-breaking antioxidant, both on its own and in combination with α-tocopherol and ascorbic palmitate in binary and ternary mixtures (Figure 3) [21]. As for the α,β-unsaturated ketones (*Z*)-**3** and (*Z*)-**4**, no synthetic methods have been reported for the preparation of the corresponding symmetric dimers **5** and **6** with (*Z*)-(*Z*) geometries.

## 2. Results and Discussion

In this paper, we present the first detailed study on the geometrical photoisomerization of curcumin-like α,β-unsaturated ketones (*E*)-**3**, (*E*)-**4**, (*E*)-(*E*)-**5**, and (*E*)-(*E*)-**6**. The structures of all new compounds were determined based on spectroscopic data. The ^1^H-NMR data indicated that all molecules underwent partial (*E*)-to-(*Z*) isomerization upon UV light at 254 nm. No isomerization was observed at 365 nm. The experiments were conducted in deuterated solvent solutions directly within an NMR tube, enabling the rapid monitoring of reaction progress and eliminating the need for any workup procedures. Isomeric ratios were determined by ^1^H-NMR over time.

### 2.1. Photoisomerization Studies

**Figure 4:**
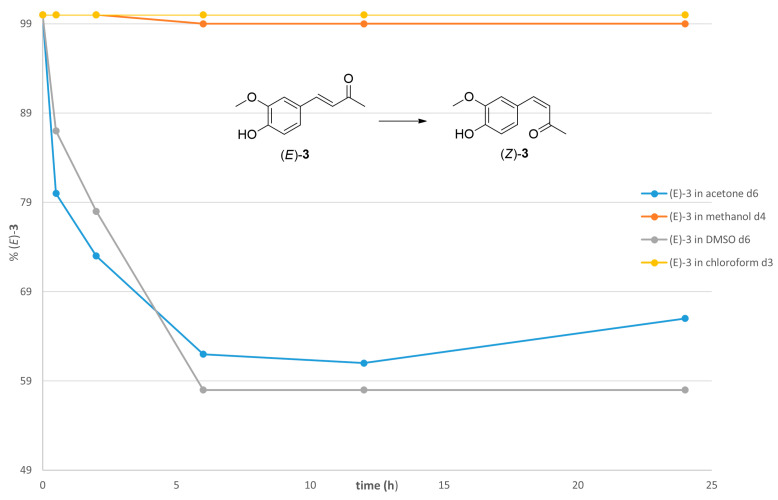
(*E*)-to-(*Z*) conversion rate of compound (*E*)-**3** upon irradiation at 254 nm in deuterated solvents: acetone, methanol, DMSO, and chloroform.

The impact of different deuterated solvents on the efficiency of the (*E*)-to-(*Z*) photoisomerization process was initially explored. Acetone-d_6_ and DMSO-d_6_ exhibited the most substantial geometrical conversion among all the deuterated solvents tested. After 24 h of UV light exposure (254 nm), 40.0% of (*E*)-**3** was converted into the corresponding isomer (*Z*)-**3** in deuterated acetone (Figure 4). Long-term stability tests did not show any significant changes. The absence of reactivity for compound (*E*)-**3** in deuterated methanol is consistent with Moni et al. [18], who did not detect any isomerization of compound (*E*)-**3** using deuterated methanol as the solvent.

**Figure 5:**
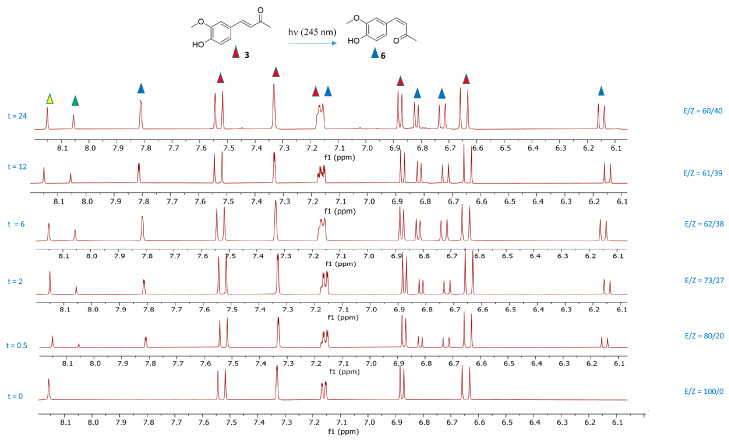
^1^H-NMR spectra of aromatic and olefinic protons of (*E*)-**3** and (*Z*)-**3** in deuterated acetone recorded after irradiation of (*E*)-**3** at 254 nm for 0, 0.5, 2, 6, 12, and 24 h. The red triangles indicate the aromatic and olefinic signals of the isomer (*E*)-**3**, while the blue triangles indicate the aromatic and olefinic signals of the isomer (*Z*)-**3**. The yellow triangle indicates the OH signal of the isomer (*E*)-**3**. The green triangle indicates the OH signal of the isomer (*Z*)-**3**. Integrations showing the (*E*)/(*Z*) ratio at different times are reported.

The spectroscopic analysis was mainly focused on determining the ratio of the integration values of the diagnostic olefinic protons, which served as key indicators of the compound’s structural features (Figure 5). For compound (*E*)-**3**, the olefinic protons appeared at 7.52 and 6.64 ppm, while for compound (*Z*)-**3**, the corresponding peaks were observed at 6.73 and 6.14 ppm.

**Figure 6:**
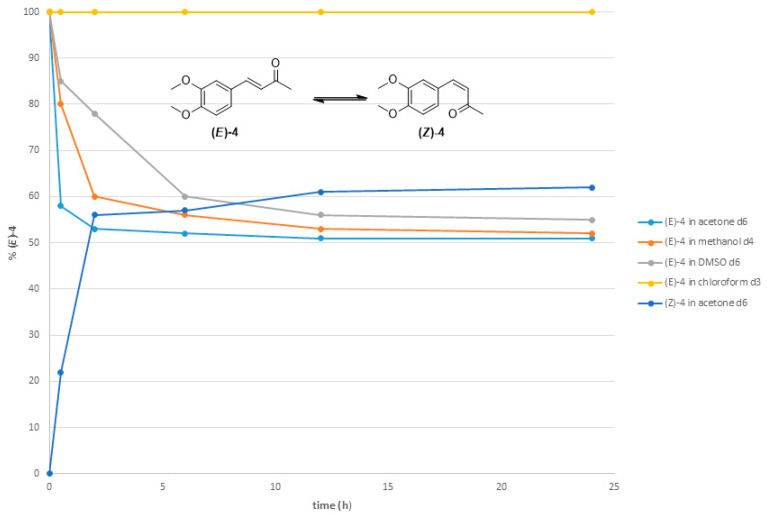
(*E*)-to-(*Z*) conversion rate of compound (*E*)-**4** after irradiation at 254 nm in deuterated solvents: acetone, methanol, DMSO, and chloroform. (*Z*)-to-(*E*) conversion rate of compound (*Z*)-**4** at 254 nm in deuterated acetone.

Kinetic photoisomerization studies and the geometrical stability of the pure compounds (*E*)-**4** and (*Z*)-**4** in different deuterated solvents showed that the conversion rates of the two isomers in acetone, DMSO, and methanol rapidly declined after 2 h and stabilized after 24 h (Figure 6). Specifically, the conversion of (*E*)-**4** to (*Z*)-**4** in deuterated acetone stabilized at 51%, while the conversion of (*Z*)-**4** to (*E*)-**4** in the same solvent stabilized at 61% after 24 h. These results show a predominance of the thermodynamically more stable isomer (*E*)-**4**. The photoisomerization of (*E*)-**4** at 254 nm does not occur in deuterated chloroform. The kinetic studies were extended to 36 h to ensure that photostationary states had been reached after 24 h.

**Figure 7:**
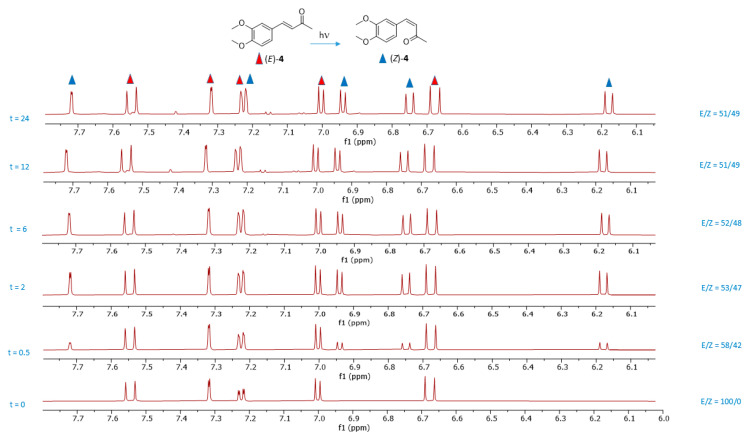
^1^H-NMR spectra of aromatic and olefinic protons of (*E*)-**4** and (*Z*)-**4** in deuterated acetone recorded after irradiation of (*E*)-**4** at 254 nm over time intervals of 0, 0.5, 2, 6, 12, and 24 h. The red triangles indicate the signals of the isomer (*E*)-**4**, while the blue triangles indicate the signals of the isomer (*Z*)-**4**. The integration showing the (*E*)/(*Z*) ratio is reported.

**Figure 8:**
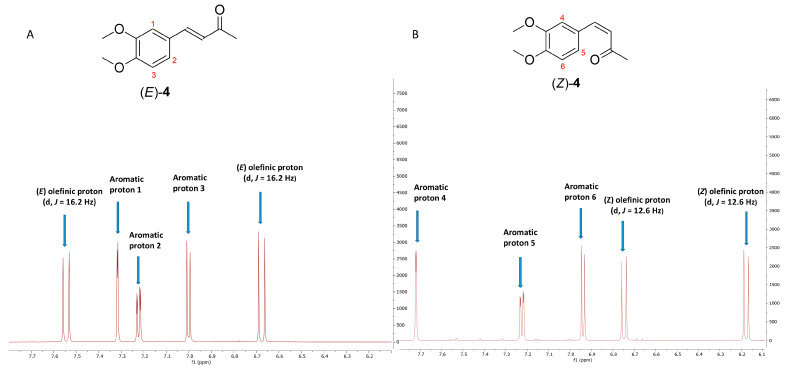
^1^H-NMR spectra of aromatic and olefinic protons in deuterated acetone of compound (*E*)-**4** (**A**) and compound (*Z*)-**4** (**B**). Both compounds were isolated with a purity > 95%.

The spectroscopic analysis centered on the ratio of the integration values of the diagnostic olefinic proton peaks (Figure 7). The ¹H-NMR spectrum of compound (*E*)-**4** (Figure 8A) displayed two signals for olefinic protons at 7.54 ppm (1H, d, *J* = 16.2 Hz) and 6.68 ppm (1H, d, *J* = 16.2 Hz) and for ABX aromatic protons at 7.31 ppm (1H, d, J = 2.4 Hz, H-3), 7.22 ppm (1H, dd, *J* = 8.4, 2.4 Hz, H-2), and 7.02 ppm (1H, d, *J* = 8.4 Hz, H-1). After 24 h of UV exposure at 254 nm (Figure 8B), a second set of five signals, indicative of compound (*Z*)-**4**, emerged: two olefinic protons at 6.74 ppm (1H, d, *J* = 12.6 Hz) and 6.17 ppm (1H, d, *J* = 12.6 Hz) and ABX aromatic protons at 7.71 ppm (1H, d, *J* = 2.4 Hz, H-4), 7.22 ppm (1H, dd, *J* = 8.4, 2.4 Hz, H-5), and 6.94 ppm (1H, d, *J* = 8.4 Hz, H-6). A noticeable downfield shift is observed in the (*E*)-isomer (*Z*)-**4** for one of the two olefinic protons and the aromatic proton H-4.

**Scheme 1:**
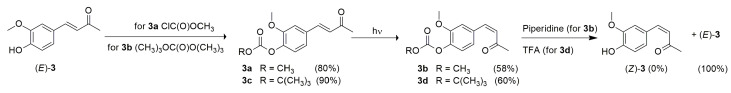
Attempted synthesis of compound (*Z*)-**3** by the protection of the hydroxyl group of compound (*E*)-**3** using chloro methyl carbonate or di *tert*-butyl dicarbonate, followed by photoisomerization of the resulting carbonates, and subsequent hydroxyl deprotection.

While compound (*Z*)-**4** was easily purified by flash chromatography, the derivative (*Z*)-**3** proved difficult to isolate because the crude reaction mixture consistently displayed one TLC spot under various elution conditions. Flash chromatography only yielded the starting material (*E*)-**3**, suggesting rapid (*Z*)-to-(*E*) retro-isomerization. To address this issue, the hydroxyl group of DHZ was protected as a methyl carbonate, forming the (*E*) derivative **3a**. Subsequent UV irradiation at 254 nm in deuterated acetone for 12 h followed by flash chromatography successfully isolated the (*Z*) derivative *O*-methyl carbonate **3b** in good yield. Unfortunately, deprotection with piperidine only gave (*E*)-**3** in quantitative yield, showing the instability of isomer (*Z*)-**3** in these conditions (Scheme 1).

The aromatic hydroxyl group was then protected using an O-*tert*-butyl dicarbonate (BOC) group, which typically facilitates faster deprotection under milder conditions. Although the photoisomerization of compound **3c** was successful and the (*Z*) derivative **3d** was isolated, subsequent deprotection with TFA only yielded the (*E*)-**3** isomer once again, with an almost quantitative yield.

**Scheme 2:**
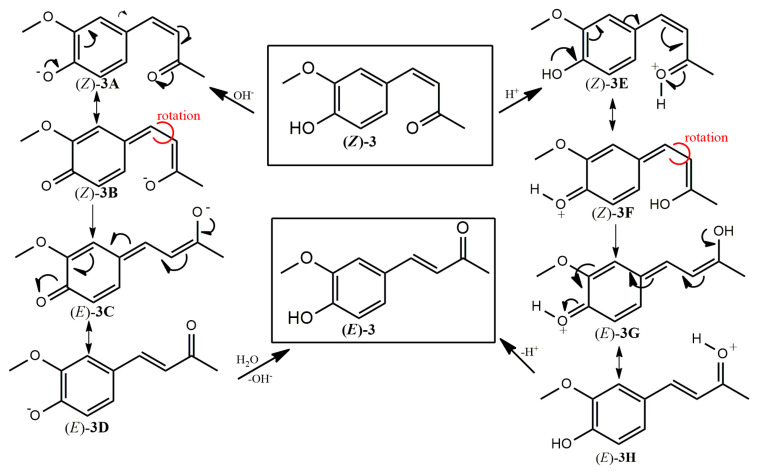
Proposed mechanism of (*Z*)-to-(*E*) retro-isomerization of compound (*Z*)-**3** in basic or acidic media. The red curves represent a rotation around the specified bonds.

We hypothesize that the extreme instability of the DHZ isomer (*Z*)-**3** in both acidic and basic environments is primarily due to the hydroxyl group attached to the aromatic ring in the para position relative to the α,β-unsaturated chain. This group promotes the formation of quinonoid structures and facilitates efficient charge delocalization throughout the molecule. The mesomeric forms (*Z*)-**3B** and (*Z*)-**3F** exhibit greater steric strain than the corresponding forms (*E*)-**3C** and (*E*)-**3G**, making the latter more influential in driving the retro-isomerization reaction, leading exclusively to isomer (*E*)-**3** (Scheme 2).

Compounds OMe DHZ (*Z*)-**4**, **3b,** and **3d** (Scheme 1) exhibit significantly greater geometric stability compared to (*Z*)-**3**. This pronounced difference in stability is likely due to variations in the electron-donating abilities of the substituents in the aromatic hydroxyl group. The methoxy group is a moderate electron donor, while the carbonate group is an electron-withdrawing system, which prevents the formation of resonance structures. Therefore, retro-isomerization through diene bond rotation, as hypothesized in (*Z*)-**3B** and (*Z*)-**3F,** is not feasible.

**Scheme 3:**
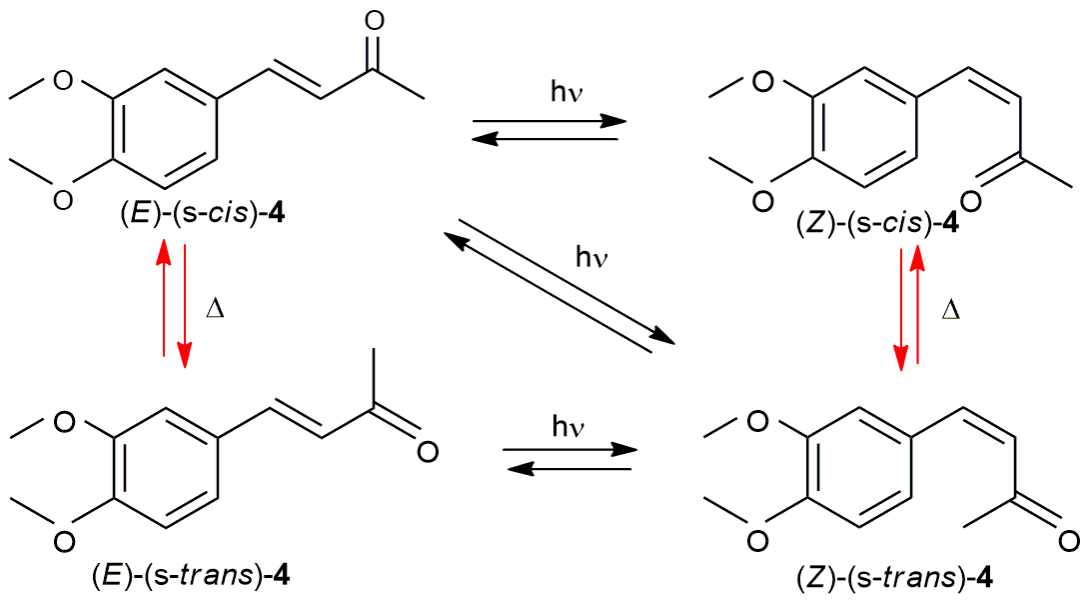
Configurational and conformational isomerization of compound **4**. Red arrows represent thermal (Δ) isomerization, black arrows represent photochemical (hν) isomerization.

An intriguing aspect of the photochemistry of α,β-unsaturated ketones is the potential for changes in both the configuration of the C=C bond and the conformation of the enone system. As illustrated in Scheme 3, with black arrows representing photochemical processes and red arrows representing thermal processes, two possible mechanisms could account for both the configurational and conformational isomerization of compound **4**. As a result, four theoretical isomers can be produced: (*E*)-(s-*cis*)-**4**, (*Z*)-(s-*cis*)-**4**, (*Z*)-(s-*trans*)-**4**, and (*E*)-(s-*trans*)-**4**.

**Figure 9:**
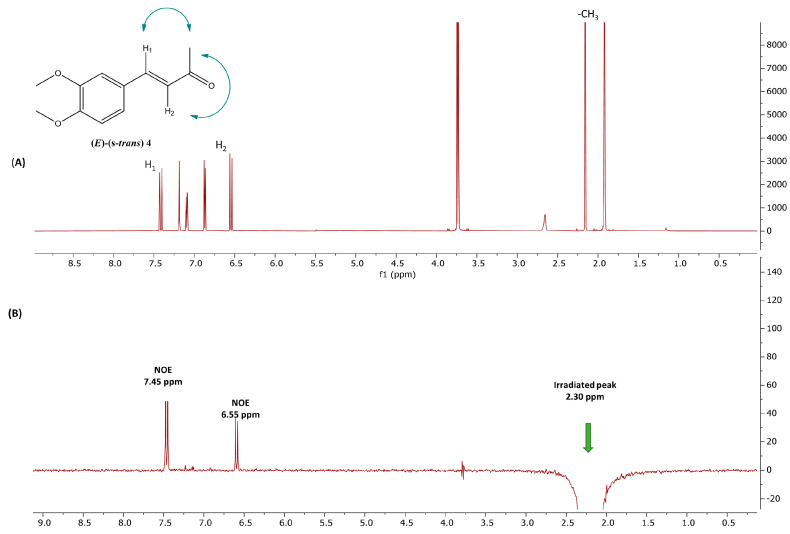
(**A**) ^1^H-NMR spectrum of compound (*E*)-(s-*trans*)-**4** in deuterated acetone; (**B**) 1D NOE ^1^H-NMR spectrum of (*E*)-(s-*trans*)-**4** in deuterated acetone after saturation of methyl group (2.30 ppm). Blue arrows illustrate NOE interactions between the highlighted protons.

The molecular structure of compound (*E*)-**4** was recently determined through X-ray crystallography, revealing an almost planar configuration with an (*E*) arrangement around the C=C double bond and an (s-*trans*) conformation between the C=C double bond and the carbonyl group [22]. In our study, the NOE (Nuclear Overhauser Effect) ^1^H-NMR technique was employed to confirm this geometry in solution (Figure 9). NOE interactions were observed between the methyl group and the olefinic protons H_1_ (7.45 ppm) and H_2_ (6.55 ppm). These NOE interactions provide compelling evidence that, in solution, the compound adopts the same (*E*)-(s-*trans*) conformation as seen in the crystallographic structure, validating the spatial arrangement of the molecule in its stable form.

**Figure 10:**
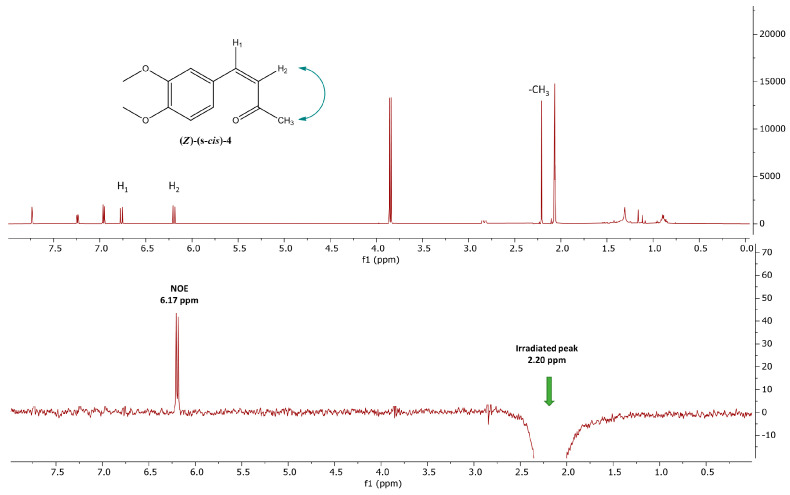
(**A**) ^1^H-NMR spectrum of compound (*Z*)-(s-*cis*)-**4** in deuterated acetone; (**B**) 1D NOE ^1^H-NMR spectrum of (*Z*)-(s-*cis*)-**4** in deuterated acetone after saturation of methyl group (2.20 ppm). The blue arrows illustrate NOE interactions between the highlighted protons.

We also studied the conformational geometry of compound (*Z*)-**4**. The NOE spectrum analysis reveals a clear interaction between the methyl group and the olefinic proton H_2_ (Figure 10). This observation, further supported by in silico energy minimization experiments (see Section 2.4), allows us to assign the (s-*cis*) conformation to compound (*Z*)-**4**.

**Figure 11:**
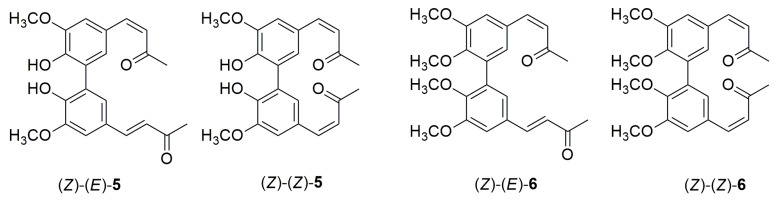
Chemical structures of compounds (*Z*)-(*E*)-**5,** (*Z*)-(*Z*)-**5,** (*Z*)-(*E*)-**6**, and (*Z*)-(*Z*)-**6**.

So far, no synthetic method has been documented for converting the (*E*)-(*E*) isomers **5** and **6** into symmetric dimers (*Z*)-(*Z*)-**5** and (*Z*)-(*Z*)-**6**, or asymmetric dimers (*Z*)-(*E*)-**5** and (*Z*)-(*E*)-**6**. (Figure 11). In this study, photoisomerization experiments were conducted directly in NMR tubes under UV light at 254 nm, using deuterated acetone as the solvent. After 12 h of irradiation, the geometrical isomerization conversions were determined by ¹H-NMR spectroscopy.

**Figure 12:**
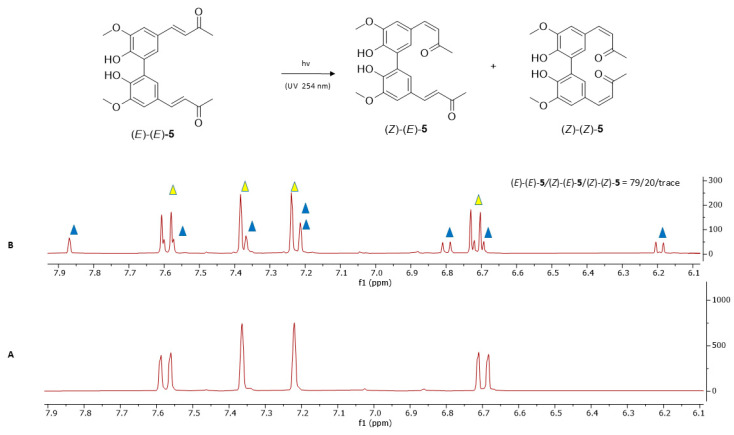
^1^H-NMR analysis of compound (*E*)-(*E*)-**5** photoisomerization in deuterated acetone after 12 h of irradiation at 254 nm. (**A**) displays the aromatic and olefinic signals of the starting material (*E*)-(*E*)-**5**. (**B**) shows the ^1^H-NMR spectrum of the reaction mixture containing compounds (*E*)-(*E*)-**5** and (*Z*)-(*E*)-**5** after photoisomerization (yellow triangles indicate the signals of the starting compound (*E*)-(*E*)-**5** and blue triangles mark the signals of the mono-isomerized (*E*)-(*Z*) isomer **5**).

**Figure 13:**
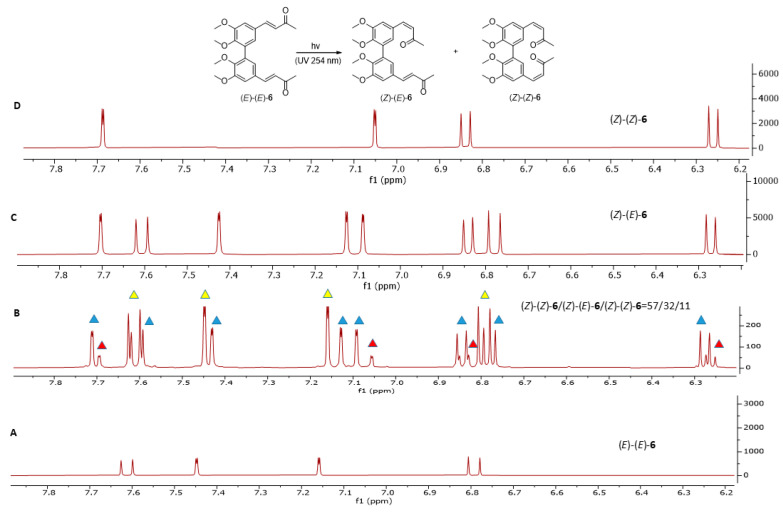
^1^H-NMR analysis of compound (*E*)-(*E*)-**6** photoisomerization in deuterated acetone after 12 h of irradiation at 254 nm. (**A**) displays the aromatic and olefinic signals of the starting material (*E*)-(*E*)-**6**. (**B**) shows the ^1^H-NMR spectrum of the crude reaction mixture after photoisomerization (red triangles denote the aromatic and olefinic signals of (*Z*)-(*Z*)-**6**, blue triangles mark the signals of mono-isomerized (*Z*)-(*E*)-**6**, and yellow triangles indicate the signals of the starting compound (*E*)-(*E*)-**6**). The aromatic and olefinic regions of purified (*Z*)-(*E*)-**6** and (*Z*)-(*Z*)-**6**, obtained by flash chromatography, are presented in (**C**) and (**D**), respectively.

Both dimers (*E*)-(*E*)-**5** and (*E*)-(*E*)-**6** predominantly underwent geometrical isomerization to form mixed (*E*)-(*Z*) isomers, while only one of the two α,β-unsaturated groups exhibited (*E*)-to-(*Z*) isomerization. Specifically, compound (*E*)-(*E*)-**5** produced dimer (*Z*)-(*E*)-**5** with a 20% conversion (Figure 12), while dimer (*E*)-(*E*)-**6** underwent mono double-bond isomerization to yield compound (*Z*)-(*E*)-**6** in 32% yield (Figure 13).

Under these conditions, dimers (*Z*)-(*Z*)-**5** and (*Z*)-(*Z*)-**6** were obtained only in trace and in 11% yield, respectively. Long-term UV light exposure experiments did not show any significant changes. Asymmetric dimer (*Z*)-(*E*)-**5**, like compound (*Z*)-**3**, is highly unstable on silica gel, making its flash chromatography purification unviable.

The (*E*)-to-(*Z*) photoisomerization reactions of the α,β-unsaturated ketones **3**, **4**, **5**, and **6** discussed in this study yield chemical efficiencies in the range of 30–50%. These relatively modest yields are consistent with those reported in the literature for the photochemical isomerization of α,β-unsaturated acids, amides, or esters [18,23]. This synthetic approach offers notable advantages over hypothetical or alternative methods, such as the stereoselective Horner–Wadsworth–Emmons reaction [24], due to its superior atom economy, straightforward procedure, and real-time monitoring of the isomerization process. This synthetic procedure can be performed with (*E*) compound concentrations ranging from 50 to 200 mM, while ensuring a constant (*E*)/(*Z*) isomeric ratio in the photostationary state.

### 2.2. Thermal Stability of (Z) Isomers

**Scheme 4:**
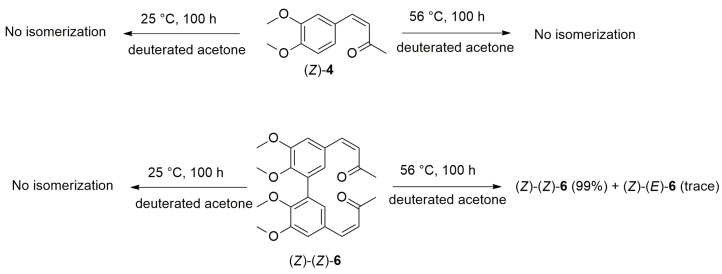
Thermal stabilities of compounds (*Z*)-**4** and (*Z*)-(*Z*)-**6**.

We performed experiments in the dark to assess the configurational thermal stability of pure compound (*Z*)-**4** and of its dimer (*Z*)-(*Z*)-**6** (Scheme 4). Each compound, in deuterated acetone, was heated at 25 °C and 56 °C for 100 h, and the (*Z*)/(*E*) ratio was monitored using ¹H-NMR spectroscopy.

Compounds (*Z*)-**4** and (*Z*)-(*Z*)-**6** demonstrate high thermal stability at 25 °C, maintaining 100% and 99% of their (*Z*) or (*Z*)-(*Z*) isomeric configuration, respectively, after 100 h at 56 °C. In the case of compound (*Z*)-(*Z*)-**6**, only partial mono-retro isomerization to the (*E*)-(*Z*)-**6** isomer was observed (trace of the compound), with no evidence of the concurrent isomerization of the two double bonds in the molecular structure.

### 2.3. UV-Vis Studies

**Figure 14:**
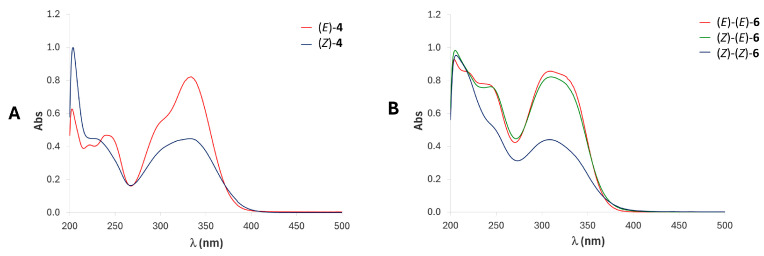
(**A**) Comparison of the UV spectra of compounds (*E*)-**4** (red) and (*Z*)-**4** (blue) at 35 μM in acetone. (**B**) Comparison of the UV spectra of compounds (*E*)-(*E*)-**6** (red), (*Z*)-(*E*)-**6** (green), and (*Z*)-(*Z*)-**6** (blue) at 35 μM in acetone.

The geometrical (*E*)-to-(*Z*) isomerization of compound (*E*)-**4** was also confirmed by UV-Vis spectroscopy. The spectra of the pure compounds (*E*)-**4** and (*Z*)-**4** dissolved in acetone (Figure 14A) exhibit intense absorption in the 260–360 nm range, with molar extinction coefficients of 10^4^ M cm^−1^, typical of highly conjugated systems. At λ_max_ of 334 nm, the absorption of (*E*)-**4** is higher than that of the corresponding (*Z*) isomer. After isomerization, band broadening at shorter wavelengths is observed, along with a slight bathochromic shift (λ_max_ 338 nm for (*Z*)-**4**). Additional intense bands appear below 260 nm, with significant absorption differences between the two compounds. In particular, a strong peak is observed in the spectra of (*E*)-**4** at 202 nm, which increases significantly upon isomerization to (*Z*), exhibiting a modest red shift (λ_max_ = 204 nm). At λ = 254 nm, the operating photoisomerization wavelength, both isomers absorb similarly (Abs 0.36 vs. 0.27), which explains the partial isomerization between them, resulting in similar E/Z ratio values once the photostationary states are reached.

UV-Vis measurements were also carried out to elucidate the conversion of dimer (*E*)-(*E*)-**6** to (*Z*)-(*E*)-**6** and (*Z*)-(*Z*)-**6**. The spectra of the isolated compounds dissolved in acetone (Figure 14B) resemble the behavior of their corresponding monomers, showing a strong absorption for (*E*)-(*E*)-**6** in the 280–360 nm region (λ_max_ 309 nm), which drastically decreases in intensity after the isomerization to (*Z*)-(*Z*)-**6**. Notably, the (*Z*)-(*Z*)-**6** isomer shows an intense band at 206 nm. Compound (*E*)-(*Z*)-**6** exhibits a spectrum that is intermediate between those of the (*E*)-(*E*)-**6** and (*Z*)-(*Z*)-**6** isomers, supporting its mixed configuration.

### 2.4. In Silico Investigation

In silico studies were carried out to elucidate the structural and electronic properties of (*E*)-**4** and (*Z*)-**4** isomers. First, the equilibrium between s-*cis* and s-*trans* conformers of both compounds was studied by DFT calculations, as described in the experimental section. The calculations were performed in acetone solution and the results are collected in Table S1. It can be noticed that the values of Gibbs energy calculated for both (*E*) (s-*cis*)-**4** and (*E*) (s-*trans*)-**4** isomers are quite close to each other, with differences of 0.4 kcal/mol; therefore, the compounds may be present in solution as a mixture of the s-*trans* and s-*cis* conformations. Considering that the NMR data indicate that (s-*trans*) conformation is retained in acetone solution, only the structure of (*E*)-(s-*trans*) **4** was considered for the UV-Vis spectra simulation. The energy of the two (*Z*) conformers differs by 2.12 kcal/mol, with the s-*cis* form being the more stable configuration, corroborating the results obtained by ^1^H-NMR studies (Figure 10).

**Figure 15:**
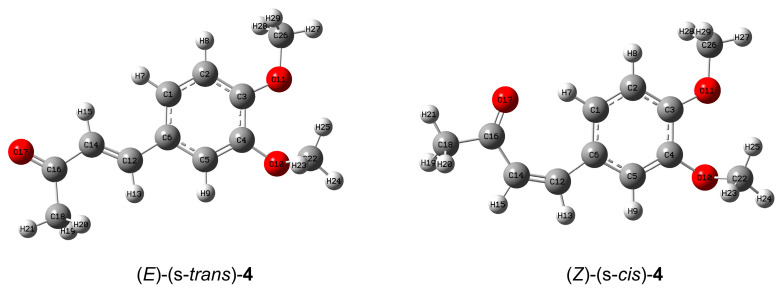
Optimized structures of the most stable isomers (*E*)-(s-*trans*)-**4** and (*Z*)-(s-*cis*)-**4**, calculated at B3LYP/6311g(d,p) level of theory in acetone.

The optimized structures of (*E*)-(s-*trans*)-**4** and (*Z*)-(s-*cis*)-**4** are shown in Figure 15. It can be noticed that both compounds assume a planar conformation, with the (*Z*)-(s-*cis*)-**4** isomer showing a more pronounced steric hindrance in the α,β-unsaturated chain compared to the more linear (*E*)-(s-*trans*) isomer, leading to a bent geometry. The most significant geometric differences are reflected in the bond angles (Table S2). In the (*E*)-(s-*trans*)-**4** structure, where the bulky substituents are on opposite sides of the double bond, the bond angles are likely closer to the ideal values for sp^2^-hybridized atoms, around 120°. On the other hand, in the (*Z*) conformation, the molecule adjusts to minimize repulsion, leading to distorted bond angles, particularly around the double bond and adjacent atoms with bond angles that deviate from the ideal 120° due to steric strain, pushing the groups apart.

**Figure 16:**
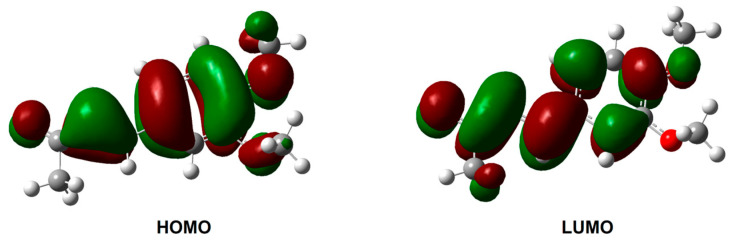
HOMO and LUMO orbitals of (*E*)-(s-*trans*)-**4** computed by TD-DFT.

**Figure 17:**
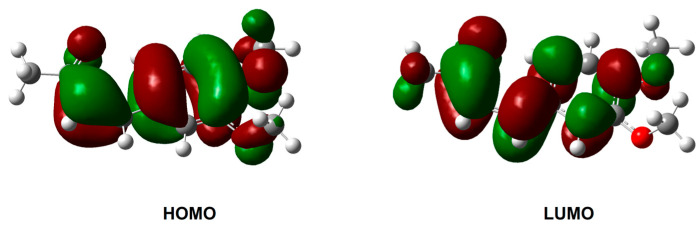
HOMO and LUMO orbitals of (*Z*)-(s-*cis*)-**4** computed by TD-DFT.

As mentioned in Section 2.3, the differences in the UV-Vis spectra of the (*E*)-**4** and (*Z*)-**4** isomers are likely due to their distinct electronic environments. The TD-DFT calculations performed on the (*E*)-(s-*trans*)-**4** and (*Z*)-(s-*cis*)-**4** structures allow the assignment of the main electronic transitions and support the interpretation of the experimental absorption spectra. Wavelength (λ), oscillator strength (ƒ), and major contributions to the calculated transitions are given in Tables S3 and S4. The highest absorption is assigned to the HOMO → LUMO contribution for both (*E*)-(s-*trans*)-**4** and (*Z*)-(s-*cis*)-**4**, and corresponds to a π → π * transition. Frontier molecular orbitals are depicted in Figure 16 and Figure 17. The slight red shift observed in the UV-Vis experimental spectra after the (*E*)-to-(*Z*) isomerization is confirmed by the calculated λ values (333.51 nm and 340.24, respectively); additionally, the higher oscillator strength values for the (*E*) isomer (0.6580) compared to the (*Z*) isomer (0.5350) support the experimental evidence discussed above. The more pronounced absorption of (*E*)-**4** in this region is possibly due to a more linear arrangement and reduced steric strain compared to the (*Z*) configuration, which facilitate a better overlap of π-orbitals, allowing greater conjugation.

The computed transitions also reflect the behavior of the two compounds at low wavelength: two strong transitions at 248.29 and 238.86 nm attributed to H → L + 1 (59%) and H-3 → L (73%), obtained for (*E*)-(s-*trans*)-**4**, are shifted at lower wavelength in the computed spectra of the Z isomer (242.37 and 230.93 nm). In addition, the absorption band calculated for the (*Z*)-(s-*cis*)-**4** isomer at 203.00 nm exhibits a higher oscillator strength compared to the corresponding band for the (*E*) isomer, confirming the higher absorption observed in the experimental spectra.

## 3. Materials and Methods

### 3.1. General

Reagents were obtained from Sigma Aldrich, Munich, Germany, and were used without further purification. ^1^H-NMR and ^13^C-NMR spectra were recorded in deuterated CDCl_3_ or DMSO or MeOH or acetone solution at 600 and 150 MHz, respectively, with a 600 MHz NMR spectrometer Bruker Avance III HD (Palo Alto, CA, USA). Full characterization data, including copies of the ^1^H-NMR and ^13^C-NMR spectra (see Supporting Information), have been reported for all the new compounds. Chemical shifts are given in ppm (δ); multiplicities are indicated by s (singlet), d (doublet), t (triplet), q (quartet), m (multiplet) or dd (doublet of doublets). Elemental analysis was performed using an elemental analyzer model 240 C (Perkin Elmer, Waltham, MA, USA). UV-Vis spectra were recorded with a Perkin-Elmer Lambda 35 spectrophotometer. Flash chromatography was performed with silica gel 60 (230–400 mesh) (VWR, Radnor, AF, USA), eluting with an appropriate solution in the stated v:v proportions. Photoisomerizations were performed with a spectroline model ENF-240C/FE UV lamp operating at 254 nm with an intensity of 390 μ W/cm (Fischer-Scientific, Vantaa, Finland). Reactions were monitored by ^1^H-NMR and analytical thin-layer chromatography (TLC) with 0.25 mm thick silica gel plates (60 F 254) (Sigma Aldrich, Munich, Germany). The melting point was determined on a 530 apparatus (Büchi, Flawil, Switzerland) and is uncorrected. The purity of new compounds was judged to be >98% by ^1^H-NMR spectral determination.

### 3.2. General Procedure for Synthesis of (E)-2-Methoxy-4-(3-Oxobut-1-en-1-Yl) Phenyl Methyl Carbonate ***3a*** and (E)-2-Methoxy-4-(3-Oxobut-1-en-1-Yl) Phenyl Tert-Butyl Carbonate ***3c***

(*E*)-**3** (0.5 g, 2.6 mmol) was added to a solution of NaOH (0.4 g, 10 mmol) in water (10 mL) at 0 °C. The solution was stirred for 1 h at 0 °C. Chloro methyl carbonate (0.37 g, 3.9 mmol) or di *tert* butyl dicarbonate (0.85 g, 3.9 mmol) was added and the mixture was stirred at rt for 2 h. The resulting solid was filtered and washed with water (3 × 50 mL) to give **3a** or **3c** as a white solid.

**3a** (80%); mp 85–86 °C; ^1^H-NMR δ 2.32 (s, 3H), 3.86 (s, 3H), 3.92 (s, 3H), 6.80 (d, *J* = 16.2 Hz, 1H), 7.22 (d, *J* = 8.4 Hz, Ar, 1H), 7.30 (dd, *J* = 1.8, 8.4 Hz, Ar, 1H), 7.48 (d, *J* = 1.8 Hz, Ar, 1H), 7.61 (d, *J* = 16.2 Hz, 1H), ^13^C-NMR δ 27.46, 55.92, 56.41, 112.64, 122.13, 123.60, 128.42, 134.91, 142.52, 142.85, 152.25, 154.18, 206.41; Anal. Calcd. for C_13_H_14_O_5_: C, 62.39; H, 5.64. Found: C, 62.46; H, 5.61.

**3c** (90%); mp 118–119 °C; ^1^H-NMR δ 1.51 (s, 9H), 2.24 (s, 3H), 3.92 (s, 3H), 6.79 (d, *J* = 16.2 Hz, 1H), 7.16 (d, *J* = 8.4 Hz, Ar, 1H), 7.27 (dd, *J* = 1.8, 8.4 Hz, Ar, 1H), 7.47 (d, *J* = 1.8 Hz, Ar, 1H), 7.60 (d, *J* = 16.2 Hz, 1H), ^13^C-NMR δ 27.47, 27.70, 56.44, 83.64, 112.62, 122.26, 123.64, 128.30, 134.62, 142.91, 143.01, 151.76, 152.73, 197.94; Anal. Calcd. for C_16_H_20_O_5_: C, 65.74; H, 6.90. Found: C, 65.76; H, 6.91.

### 3.3. General Procedure for Photoisomerization of Compounds (E)-***3***, ***3a***, ***3c***, and (E)-***4***

An NMR tube containing a 50 mM solution of the compound (*E*)-**3** or **3a** or **3c** or (*E*)-**4** in deuterated acetone was placed between two Spectroline UV lamps operating at 254 nm with an intensity of 390 μW/cm. ^1^H-NMR spectra were registered over time (t = 0, 0.5, 2, 6, 12, and 24 h) until the photostationary state was reached. The (*E*)/(*Z*) ratios were determined by integrating the olefinic protons of isomers (*E*)-**3**, **3a**, **3c**, and (*E*)-**4**, and isomers (*Z*)-**3**, **3b**, **3d,** and (*Z*)-**4**. Compounds **3b**, **3d**, and (*Z*)-**4** were purified by flash chromatography using a 1:2 mixture of ethyl acetate/petroleum ether as an eluent to obtain yellow oils.

*(Z)-2-methoxy-4-(3-oxobut-1-en-1-yl) phenyl methyl carbonate* **3b**

**3b** (58%); ^1^H-NMR δ 2.20 (s, 3H), 3.85 (s, 3H), 3.87 (s, 3H), 6.30 (d, *J* = 12.6 Hz, 1H), 6.85 (d, *J* = 12.6 Hz, 1H), 7.15 (d, *J* = 8.4 Hz, Ar, 1H), 7.20 (dd, *J* = 1.8 Hz, 8.4 Ar, 1H), 7.65 (d, *J* = 1.8 Hz, Ar,1H), ^13^C-NMR δ 30.42, 54.98, 55.46, 114.11, 121.99, 122.85, 128.92, 134.58, 138.58, 140.80, 150.87, 153.37, 199.18; Anal. Calcd. for C_13_H_14_O_5_: C, 62.39; H, 5.64. Found: C, 62.56; H, 5.69.

*(Z)-2-methoxy-4-(3-oxobut-1-en-1-yl) phenyl tert-butyl carbonate* **3d**

**3d** (60%); ^1^H-NMR δ 1.52 (s, 9H), 2.21 (s, 3H), 3.87 (s, 3H), 6.30 (d, *J* = 12.6 Hz, 1H), 6.83 (d, *J* = 12.6 Hz, 1H), 7.12 (d, *J* = 8.4 Hz, Ar, 1H), 7.19 (dd, *J* = 1.8, 8.4 Hz, Ar, 1H), 7.60 (d, *J* = 8.4 Hz, 1H), ^13^C-NMR δ 27.69, 31.28, 56.26, 83.50, 114.92, 122.83, 123.76, 129.60, 135.05, 139.9, 141.93, 151.79, 151.81, 200.01; Anal. Calcd. for C_16_H_20_O_5_: C, 65.74; H, 6.90. Found: C, 65.79; H, 6.81.

*(Z)-4-(3,4-dimethoxyphenyl) but-3-ene-2-one (Z)-* **4**

(*Z*)-**4** (49%) yield; ^1^H-NMR δ 2.20 (s, 3H), 3.82 (s, 3H), 3.83 (s, 3H), 6.17 (d, *J* = 12.6 Hz, 1H), 6.74 (d, *J* = 12.6 Hz, 1H), 6.93 (d, *J* = 8.4 Hz, Ar, 2H), 7.23 (dd, *J* = 1.8, 8.4 Hz, Ar, 1H), 7.71 (d, *J* = 1.8 Hz, Ar, 1H); ^13^C-NMR δ 30.50, 55.99, 111.80, 114.49, 125.55, 126.81, 129.21, 141.25, 149.57, 151.75, 199.64; Anal. Calcd. for C_12_H_14_O_3_: C, 69.88; H, 6.84. Found: C, 70.00; H, 6.81.

### 3.4. General Procedure for Photoisomerization of Compounds (E)-(E)-***5*** and (E)-(E)-***6***

An NMR tube containing a 50 mM solution of compounds (*E*)-(*E*)-**5** or (*E*)-(*E*)-**6** in deuterated acetone was exposed to two Spectroline UV lamps operating at 254 nm with an intensity of 390 μW/cm^2^. After 24 h of irradiation, ¹H-NMR spectra were recorded. The ratios of the (*E*)-(*E*), (*E*)-(*Z*), and (*Z*)-(*Z*) isomers were determined by integrating the olefinic protons of compounds (*E*)-(*E*)-**5**, (*Z*)-(*E*)-**5**, and (*Z*)-(*Z*)-**5** or (*E*)-(*E*)-**6**, (*Z*)-(*E*)-**6**, and (*Z*)-(*Z*)-**6**. Compounds (*Z*)-(*E*)-**6** and (*Z*)-(*Z*)-**6** were purified by flash chromatography using a 1:2 mixture of ethyl acetate/petroleum ether as an eluent to obtain yellow oils. Compound (*Z*)-(*Z*)-**5** was obtained only in trace amounts. Compound (*Z*)-(*E*)-**5** could not be isolated via flash chromatography due to its significant geometric instability.

*(3Z,3′E)-4,4′-(5,5′,6,6′-tetramethoxy-[1,1′-biphenyl]-3,3′-diyl)bis(but-3-en-2-one) (Z)-(E)-* **6**

(*Z*)*-*(*E*)*-***6** (32%) ^1^H-NMR δ 2.21 (s, 3H), 2.30 (s, 3H), 3.67 (s, 3H), 3.69 (s, 3H), 3.92 (s, 3H), 3.97 (s, 3H), 6.25 (d, *J* = 12.6 Hz, Ar, 1H), 6.67 (d, *J* = 16.2 Hz, Ar, 1H), 6.68 (d, *J* = 12.6 Hz, Ar, 2H), 7.07 (d, *J* = 2.4 Hz, Ar, 1H), 7.10 (d, *J* = 2.4 Hz, Ar, 1H), 7.41 (d, *J* = 2.4 Hz, Ar, 1H), 7.57 (d, *J* = 16.2 Hz, Ar, 1H), 7.69 (d, *J* = 2.4 Hz, Ar, 1H); ^13^C-NMR δ 26.54, 30.50, 55.31, 55.48, 59.83, 59.87, 11.05, 113.74, 124.03, 125.64, 126.47, 127.65, 130.10, 130.73, 131.86, 132.85, 139.52, 142.69, 148.01, 148.99, 152.24, 153.18, 197.10, 199.14; Anal. Calcd. for C_24_H_26_O_6_: C, 70.23; H, 6.38. Found: C, 70.09; H, 6.41.

*(3Z,3′Z)-4,4′-(5,5′,6,6′-tetramethoxy-[1,1′-biphenyl]-3,3′-diyl)bis(but-3-en-2-one) (Z)-(Z)*-**6**

(*Z*)*-*(*Z*)*-***6** (11%) ^1^H-NMR δ 2.22 (s, 6H), 3.65 (s, 6H), 3.91 (s, 6H), 6.26 (d, *J* = 12.6 Hz, 2H), 6.82 (d, *J* = 12.6 Hz, Ar, 2H), 7.67 (d, *J* = 2.4 Hz, Ar, 2H); ^13^C-NMR δ 31.10, 55.29, 59.73, 113.60, 125.69, 127.67, 130.69, 132.05, 139.52, 148.04, 152.25; Anal. Calcd. for C_24_H_26_O_6_: C, 70.23; H, 6.38. Found: C, 70.39; H, 6.21.

### 3.5. DFT Calculations

DFT calculations were carried out with Gaussian 09 (revision C.01) [25]. The geometries of the possible isomers of (*E*)-**4** and (*Z*)-**4** were computed at the B3LYP/6-311g(d, p) level of theory, considering acetone as a solvent within the framework of the SMD model [26]. For all the simulations, the minimum was verified through frequency calculations.

The relative stabilities of (*E*)-(s-*cis*)-**4**, (*E*)-(s-*trans*)-**4**, (*Z*)-(s-*cis*)-**4**, and (*Z*)-(s-*trans*)-**4** (Scheme 3) were determined by calculating the value of Gibbs free energy in acetone solution (G_sol_). The Gibbs energy in solution for each species can be separated into electronic plus nuclear repulsion energy (E_ele_), the thermal contribution (G_therm_), and solvation energy (ΔG_solv_): G_sol_ = E_ele_ + G_therm_ + ΔG_solv_. The thermal contribution was estimated using the ideal gas model and harmonic vibrational frequencies were calculated to determine the correction due to the zero-point energy and the thermal population of the vibrational levels. Time-dependent density functional theory (TD-DFT) calculations were performed at the B3LYP/6-311g(d, p) level and used to predict excited states and calculate the electronic absorption spectra.

## 4. Conclusions

Here, we report the first study on the preparation of dehydrozingerone (*Z*)-**3** and OMe dehydrozingerone (*Z*)-**4** through the photoisomerization of their respective (*E*) isomers. Our findings show that the natural compound dehydrozingerone and its *O*-methyl derivative yielded corresponding (*Z*) isomers efficiently upon UV light irradiation at 254 nm in deuterated acetone solution. Under these conditions, the photoisomerization of the related dimeric structures (*E*)-(*E*)-**5** and (*E*)-(*E*)-**6** was incomplete, resulting primarily in mixed (*E*)-(*Z*) isomers, with minimal or no isomerization of both α,β-unsaturated chains in these compounds. Compounds (*Z*)-**4**, (*Z*)-(*E*)-**6**, and (*Z*)-(*Z*)-**6** were successfully purified by flash chromatography, while the pure forms of (*Z*)-**3** and (*Z*)-(*E*)-**5** could not be isolated due to their instability in acidic and basic environments.

The thermal geometric stability of (*Z*)-**4** and (*Z*)-(*Z*)-**6** was investigated. NOE ^1^H-NMR interactions confirmed that, in solution, (*E*)-**4** retains the (*E*)-(s-*trans*) conformation observed in its crystallographic structure, thereby validating its stable molecular arrangement. The structures of these new compounds were determined using NMR spectroscopy and were corroborated by UV-Vis and computational analyses. In silico studies provided additional insights, revealing the structural and electronic characteristics of the (*E*) and (*Z*) isomers. The calculated electronic transitions corresponded closely with the experimental data.

These findings enhance our understanding of the stability and behavior of these new compounds, providing valuable insights for potential applications.

Future studies will examine how the (*Z*) geometry in newly synthesized curcuminoid compounds may impact the biological activities traditionally associated with their (*E*) isomers.

## Data Availability

Data is contained within the article or Supplementary Materials.

## Supplementary Materials

The following supporting information can be downloaded at https://www.mdpi.com/article/10.3390/molecules29245901/s1, ^1^H and ^13^C-NMR spectra of all the products synthesized in this work. Table S1: Gibbs free energy values for the possible conformers of compounds (*E*)-**4** and (*Z*)-**4**. Table S2: Bond angles of optimized (*E*)-(s-*trans*)-**4** and (*Z*)-(s-*cis*)-**4** structures. Table S3: Experimental and calculated electronic transitions of (*E*)-(s-*trans*)-**4**. Table S4: Experimental and calculated electronic transitions of (*Z*)-(s-*cis*)-**4**. Table S5: Optimized coordinates of (*E*)-(s-*cis*)-4. Table S6: Optimized coordinates of (*E*)-(s-*trans*)-4. Table S7: Optimized coordinates of (*Z*)-(s-*cis*)-4. Table S8: Optimized coordinates of (*Z*)-(s-*trans*)-4.
