# Supplementary material for: A Study on the Photoisomerization of (E)-Dehydrozingerone, Its (E)-(E)-C₂ Symmetric Dimer, and Their O-Methylated Derivatives
Source: Molecules. 2024 Dec 13;29(24):5901. doi: 10.3390/molecules29245901 (PMC11678602; doi:10.3390/molecules29245901)
Supplement: Supplementary file 1 [file molecules-29-05901-s001.zip › molecules-3337403-supplementary.pdf]

Supplementary Materials for

A Study on the Photoisomerization of (*E*)-Dehydrozingerone, its (*E*)-(*E*)-C_2 Symmetric Dimer, and their *O*-Methylated Derivatives.

Maria Antonietta Dettori, Valeria Ugone, Davide Fabbri* and Paola Carta

Istituto di Chimica Biomolecolare, Consiglio Nazionale delle Ricerche, Traversa La Crucca 3, I–07100 Sassari, Italy. E-mail: davidegaetano.fabbri@cnr.it. Phone: +39 0792841223; Fax: +39 079 2841229

*Corresponding Author: davidegaetano.fabbri@cnr.it

Table of contents

Copies of NMR spectra pages 2-8

Table S1 page 9

Table S2 page 9

Table S3 page 9

Table S4 page 9-10

Table S5 page 10-11

Table S6 page 11-12

Table S7 page 12-13

Table S8 page 13-14

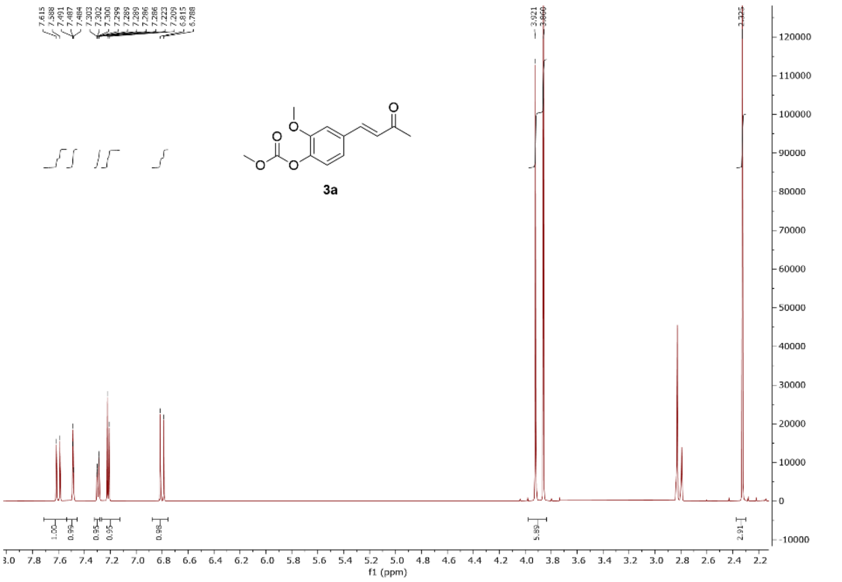

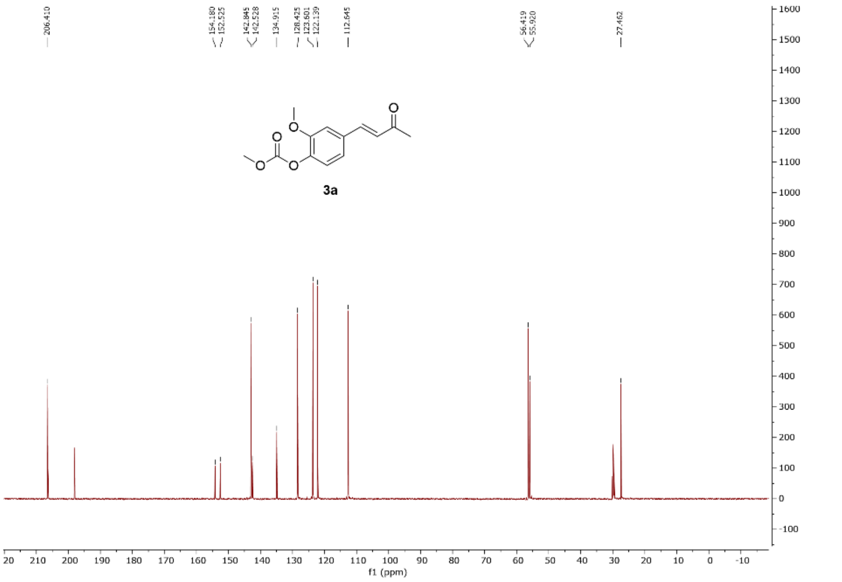

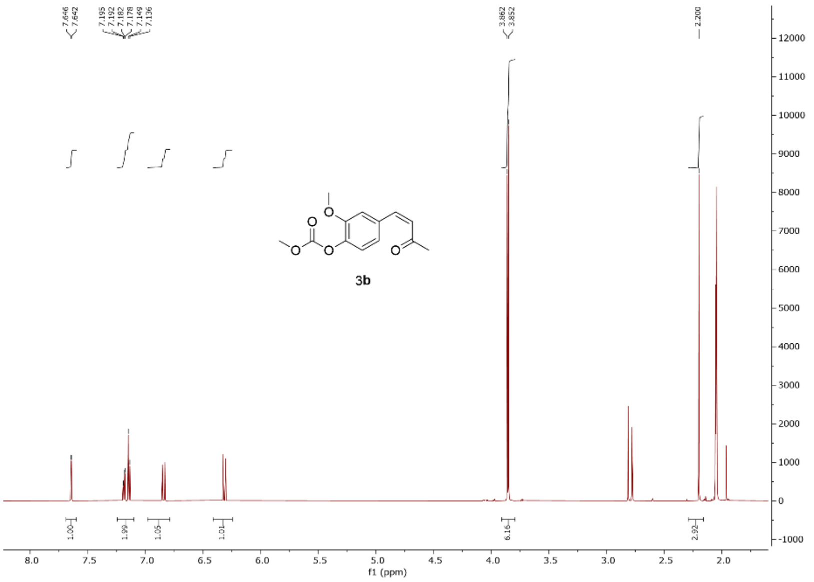
3b
f1 (ppm)

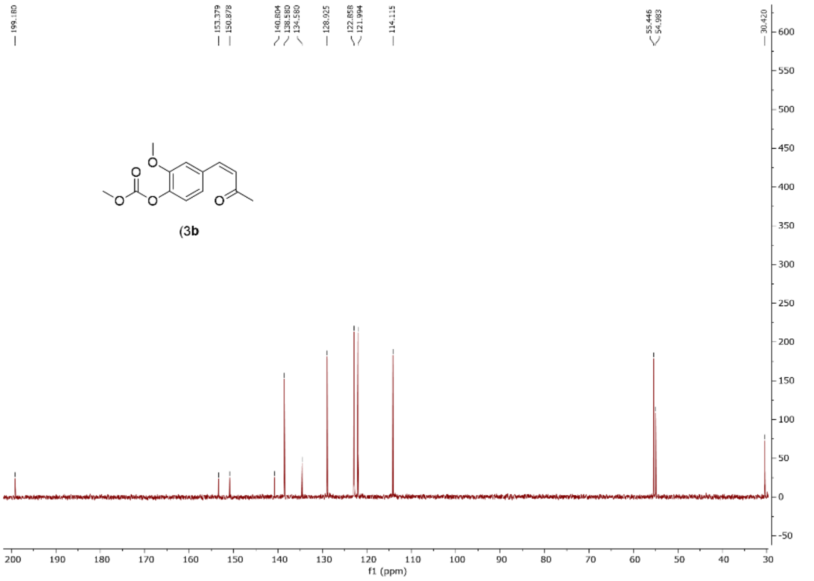
(3b
f1 (ppm)

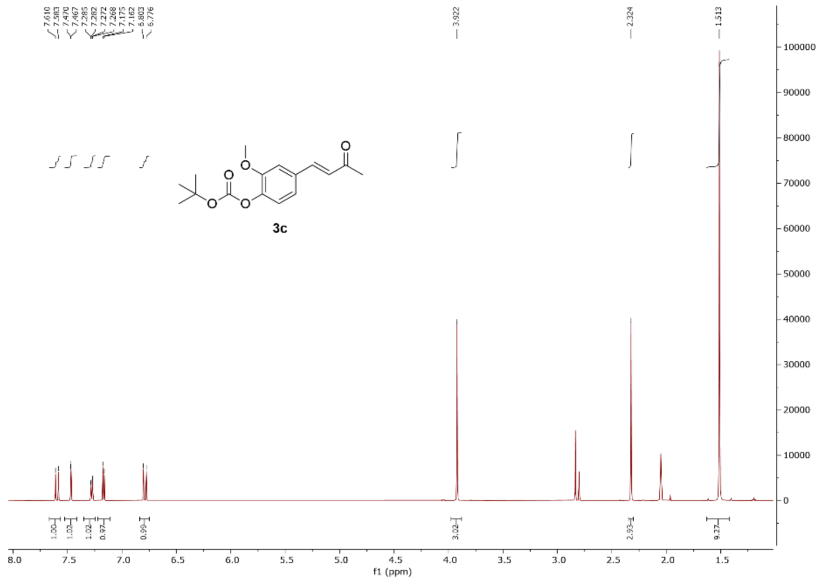

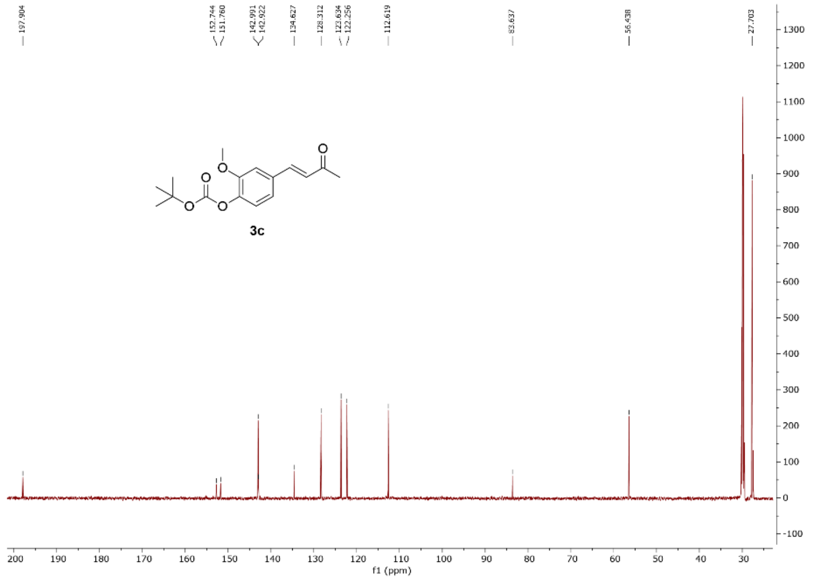

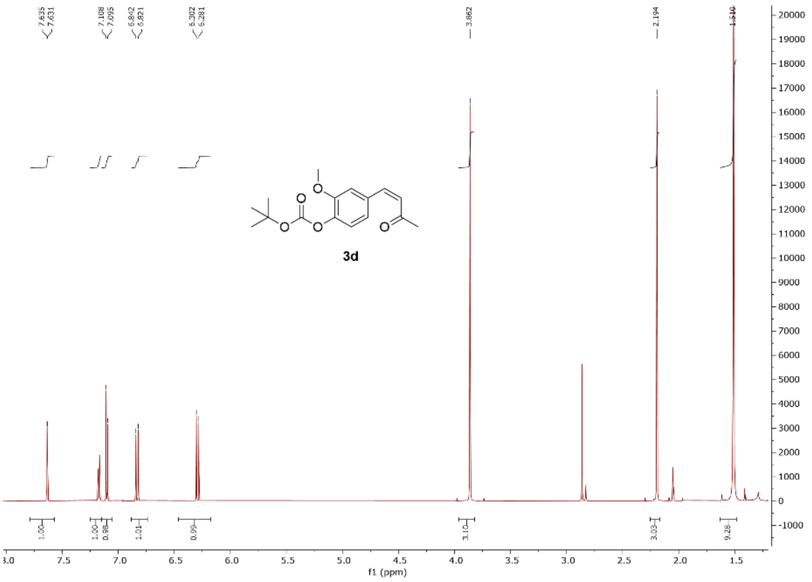
7.635
7.631
7.108
7.095
6.842
6.821
6.302
6.281
3.862
2.194
1.510
3d
1.00
1.00
0.98
1.01
0.99
3.10
3.03
9.28
f1 (ppm)

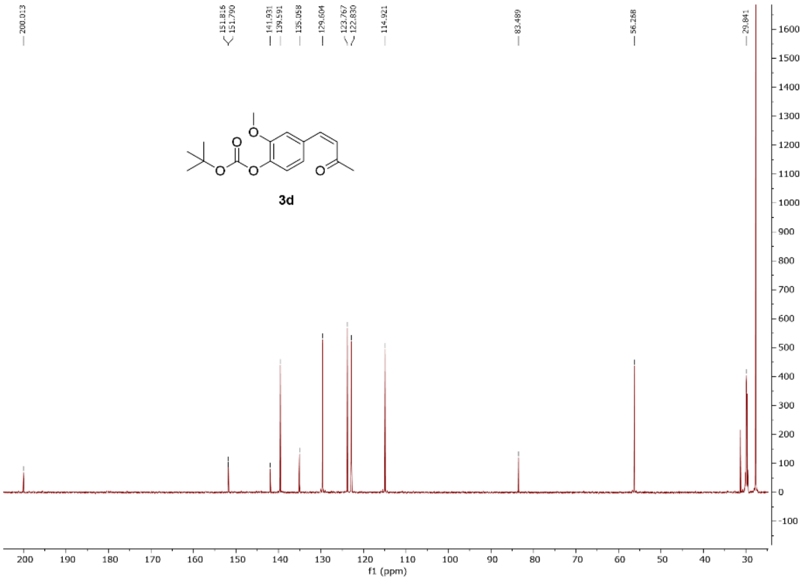
200.013
151.816
151.790
141.931
139.591
135.058
129.604
123.767
122.830
114.921
83.489
56.268
29.841
3d
f1 (ppm)

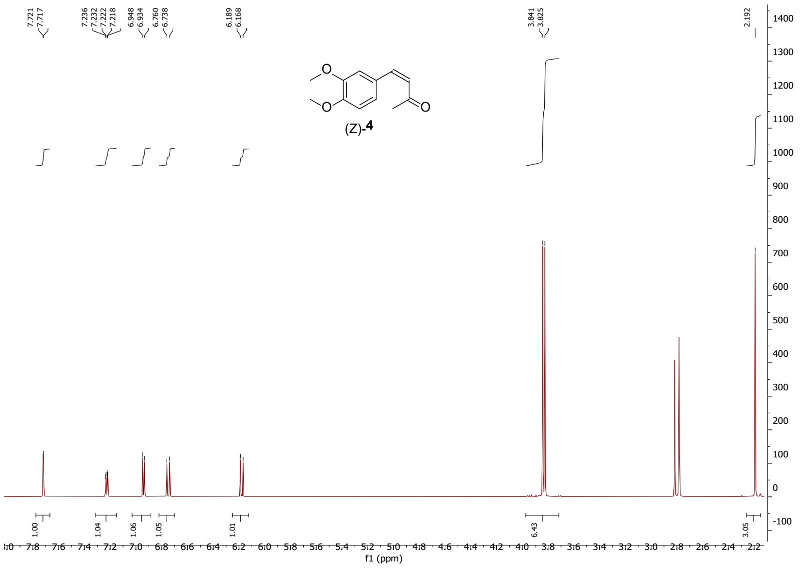

7.721
7.717
7.236
7.232
7.222
7.218
6.948
6.934
6.760
6.738
6.189
6.168
3.841
3.825
2.192
O
O
O
(Z)-4
1.00
1.04
1.06
1.05
1.01
6.43
3.05
f1 (ppm)

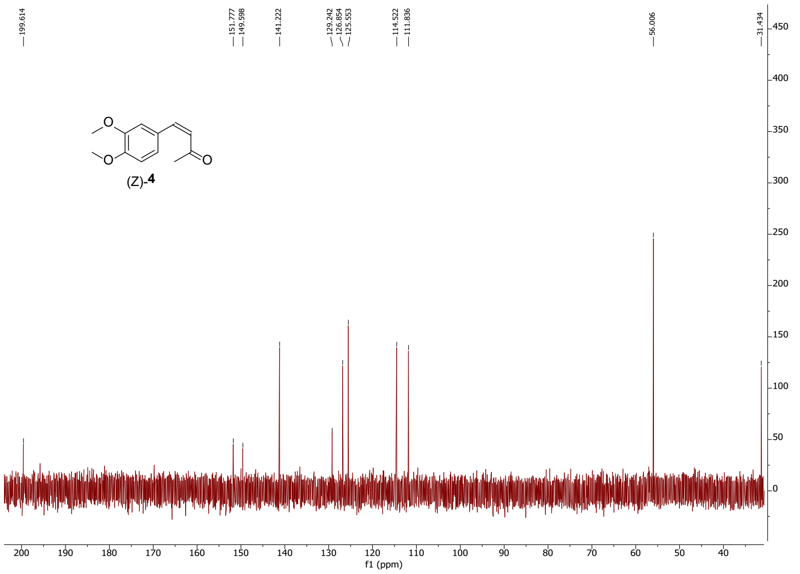

199.614
151.777
149.598
141.222
129.242
126.854
125.553
114.522
111.836
56.006
31.434
O
O
O
(Z)-4
f1 (ppm)

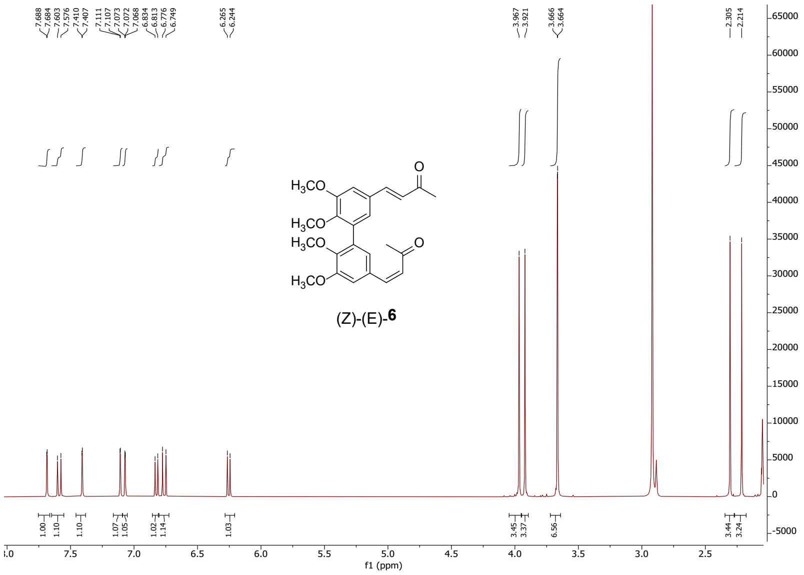

H3CO
H3CO
H3CO
H3CO
O
O
(Z)-(E)-6
f1 (ppm)

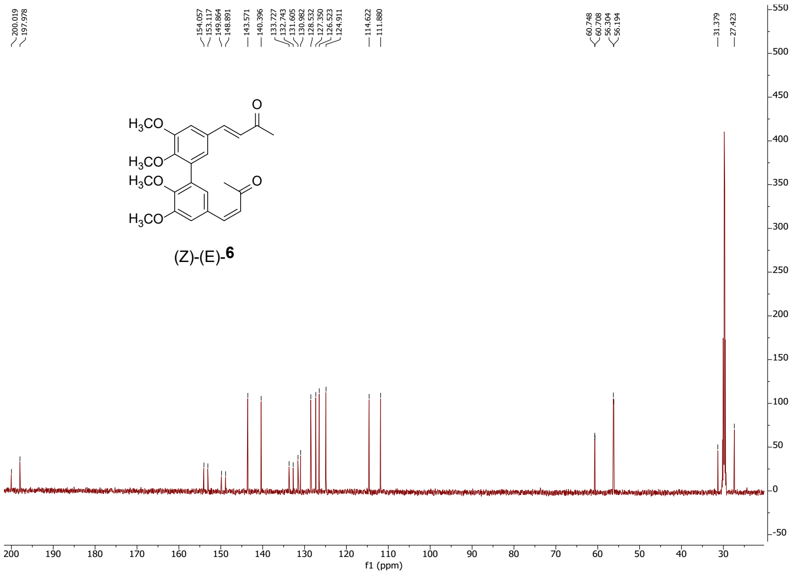

H3CO
H3CO
H3CO
H3CO
O
O
(Z)-(E)-6
f1 (ppm)

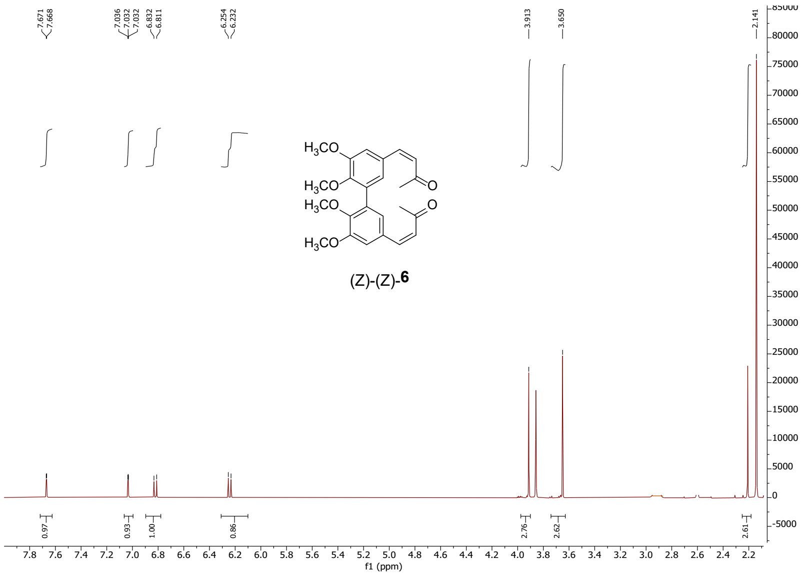

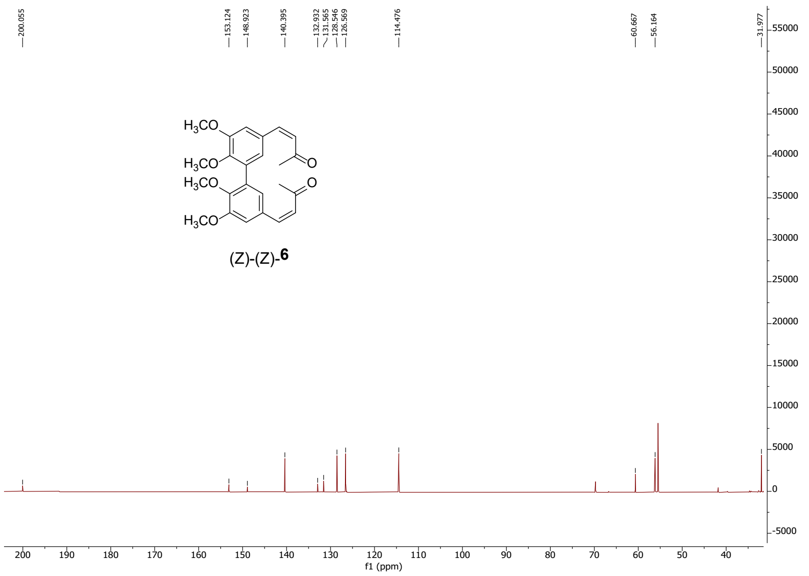

Table S1. Gibbs free energy values for the possible conformers of compounds (*E*)-**4** and (*Z*)-**4**[a].

Isomer	G_{sol} (Kcal/mol)
(*E*)-(s-*cis*)-**4**	-433828.04
(*E*)-(s-*trans*)-**4**	-433827.65
(*Z*)-(s-*cis*)-**4**	-433822.40
(*Z*)-(s-*trans*)-**4**	-433820.28

[a] Calculations performed at the B3P86/6-311g(d,p) level of theory using the SMD model for acetone.

Table S2. Bond angles of (*E*)-(s-*trans*)-**4** and (*Z*)-(s-*cis*)-**4** optimized structures.

Bond angles[a]	(E)-(s trans)-**4**	(Z)-(s-cis)-**4**
C1-C6-C12	123.6302	126.5785
C6-C12-C14	127.2574	136.2326
C12-C14-C16	125.0324	132.5989
C14-C16-O17	119.5408	125.4397

[a] For atom labels, refer to Figure 15.

Table S3. Experimental and calculated electronic transitions of (E)-(s-trans)-**4**.

**Main Transition** [a]	**f** [b]	**λ (nm)**	**λ_{exp} (nm)**
H → L (100%)	0.6580	333.51	334
H-2 → L (83%)	0.1098	288.37	300
H → L+1 (59%)<br>H-3 → L (25%)	0.2369	248.29	247
H-3 → L (73%)	0.0975	238.86	238
H-4 → L (80%)	0.1058	226.86	221
H-3 → L+1 (41%)<br>H-2 → L+1 (30%)	0.0512	202.54	203

[a] H-*n* means *n* levels lower than HOMO, and L+*n* means *n* levels upper than LUMO. [b] Oscillator strength.

Table S4. Experimental and calculated electronic transitions of (Z)-(s-cis)-**4**.

**Main Transition** [a]	**f** [b]	**λ (nm)**	**λ_{exp} (nm)**
H → L (100%)	0.5350	340.24	338
H-2 → L (91%)	0.1184	294.72	305
H-3 → L (21%)<br>H → L+1 (65%)	0.1933	242.37	

H-4 → L (82%)	0.0681	230.93	231
H → L+2 (68%)<br>H-2 → L+1 (21%)	0.1056	217.69	
H-5 → L (27%)<br>H-3 → L+1 (40%)<br>H-2 → L+1 (27%)	0.0884	203.00	204

[a] H-n means n levels lower than HOMO, and L+n means n levels upper than LUMO. [b] Oscillator strength.

Table S5: Optimized coordinates of (*E*)-(s-*cis*)-**4**

C	0.08853400	-1.27774000	0.09020300
C	-1.27108200	-1.56185000	0.07705600
C	-2.21430600	-0.53941100	-0.09261800
C	-1.76114200	0.79278300	-0.23621500
C	-0.40162300	1.05778900	-0.21726600
C	0.55645700	0.03679100	-0.06277800
H	0.78737100	-2.09578300	0.21712000
H	-1.59704700	-2.58724400	0.18909100
H	-0.08316900	2.08753000	-0.33834100
O	-2.63617600	1.82475100	-0.46178100
O	-3.55284600	-0.72799600	-0.14545600
C	1.96780800	0.39726300	-0.06778800
H	2.17821400	1.45766900	-0.19160400
C	3.03646800	-0.41451200	0.05742700
H	2.92821900	-1.48695300	0.18236700
C	4.41544400	0.12104800	0.02464500
O	4.65894700	1.31386500	-0.10446200
C	5.52524700	-0.89841800	0.15993800
H	5.42549800	-1.44001600	1.10670700

H	5.45506800	-1.64266300	-0.64060900
H	6.49705600	-0.40659100	0.11911600
C	-3.47539200	2.19068100	0.64740200
H	-2.86932000	2.47054400	1.51577800
H	-4.05088800	3.05602400	0.31812900
H	-4.15713700	1.38174000	0.91708300
C	-4.06827300	-2.05834400	-0.00867600
H	-5.15008300	-1.96151200	-0.08270000
H	-3.70538700	-2.70827300	-0.81004300
H	-3.80664200	-2.48589900	0.96351000

Table S6: Optimized coordinates of (*E*)-(s-*trans*)-**4**

C	0.06644100	-1.32313400	0.07825500
C	-1.30120900	-1.56620500	0.06423400
C	-2.21363400	-0.51527800	-0.09800500
C	-1.72084000	0.80327200	-0.23490500
C	-0.35374400	1.02723400	-0.21664800
C	0.57286900	-0.02255500	-0.06714600
H	0.74119100	-2.16193700	0.19878800
H	-1.65786300	-2.58204800	0.16927700
H	-0.00626000	2.04826500	-0.33249700
O	-2.56383300	1.86321300	-0.45259800
O	-3.55748600	-0.66409400	-0.15014600
C	1.99738400	0.29487700	-0.06697100
H	2.22162200	1.35007900	-0.19989100
C	3.02569100	-0.56577800	0.07765700
H	2.85770900	-1.62985300	0.21539900
C	4.44922600	-0.19086000	0.06787400
O	5.30381900	-1.05813400	0.21274800
C	4.84629700	1.26039000	-0.12071400

H	4.48519700	1.64432600	-1.07927000
H	4.41949900	1.88993000	0.66533900
H	5.93317500	1.33509700	-0.09094800
C	-3.39371800	2.24477200	0.65850400
H	-2.78081500	2.49499700	1.53108100
H	-3.93938100	3.13209000	0.33701800
H	-4.10232200	1.45595300	0.91789500
C	-4.11051300	-1.97970800	-0.01769100
H	-5.18977700	-1.85056900	-0.07905400
H	-3.77595700	-2.63373400	-0.82809600
H	-3.85150400	-2.42263900	0.94826900

Table S7: Optimized coordinates of (*Z*)-(s-*cis*)-**4**

C	-0.47749100	0.80324100	-0.00775200
C	0.78880000	1.37771600	0.00531400
C	1.93951300	0.58991900	-0.11400400
C	1.79601200	-0.81244200	-0.23945800
C	0.53129700	-1.36995700	-0.24954200
C	-0.64162000	-0.58639400	-0.14080700
H	-1.35284600	1.42941000	0.08256900
H	0.87688800	2.45187000	0.10164600
H	0.45785200	-2.44693600	-0.35676300
O	2.89009400	-1.62287400	-0.41406200
O	3.20370000	1.06652300	-0.13321600
C	-1.90522700	-1.31280000	-0.17342500
H	-1.74006800	-2.38116800	-0.29981600
C	-3.22404400	-0.99341400	-0.08563400
H	-3.89796200	-1.84236800	-0.15723100
C	-3.93036000	0.28739800	0.09516900
O	-3.39515000	1.38519500	0.20505400

C	-5.44199000	0.17009600	0.14446900
H	-5.81893400	-0.29825700	-0.77055500
H	-5.74617100	-0.47006600	0.97931800
H	-5.88806400	1.15757300	0.26129200
C	3.70713200	-1.81610500	0.75348400
H	3.12373000	-2.26887600	1.56250800
H	4.50381700	-2.49935500	0.45837600
H	4.14428500	-0.87540300	1.09571600
C	3.40772600	2.48116200	-0.02473700
H	4.48635500	2.62420800	-0.06343400
H	2.93978500	3.01201600	-0.85849200
H	3.02369000	2.86607000	0.92419600

Table S8: Optimized coordinates of (*Z*)-(s-*trans*)-**4**

C	-0.58192800	0.77042200	-0.62922600
C	0.67115800	1.35136000	-0.44895800
C	1.80054000	0.55521800	-0.23800500
C	1.65154100	-0.85262900	-0.21763900
C	0.40082600	-1.41371500	-0.41447400
C	-0.74671800	-0.61944100	-0.60441700
H	-1.42960100	1.41344700	-0.83192100
H	0.76489300	2.42793600	-0.49696100
H	0.32416900	-2.49576200	-0.41351100
O	2.73870000	-1.67815900	-0.08114700
O	3.06045400	1.02662800	-0.08034800
C	-2.03781000	-1.27661100	-0.82996700
H	-1.96903600	-2.20940700	-1.38703400
C	-3.28336500	-0.88583300	-0.49230000
H	-4.11155900	-1.46534400	-0.89283300
C	-3.75236500	0.24738000	0.34408300
O	-4.84580100	0.74395000	0.11120400

C	-2.96517000	0.70201200	1.55926600
H	-2.02808800	0.16876400	1.70625500
H	-2.76789900	1.77516500	1.49672000
H	-3.60519600	0.54266900	2.43302900
C	3.33815900	-1.70235500	1.22567700
H	2.60531300	-2.00616000	1.98090100
H	4.13596000	-2.44403200	1.18054200
H	3.75948300	-0.73036400	1.49078900
C	3.27667000	2.44082600	-0.14942300
H	4.34800900	2.58030800	-0.01397000
H	2.97817300	2.83860100	-1.12377900
H	2.73932300	2.96501600	0.64640400
